# Photostability of the deprotonated forms of the UV filters homosalate and octyl salicylate: molecular dissociation *versus* electron detachment following UV excitation†

**DOI:** 10.1039/d2cp01612e

**Published:** 2022-06-27

**Authors:** Natalie G. K. Wong, Conor D. Rankine, Cate S. Anstöter, Caroline E. H. Dessent

**Affiliations:** Department of Chemistry, University of York Heslington YO10 5DD UK caroline.dessent@york.ac.uk; Chemistry – School of Natural and Environmental Sciences, Newcastle University, Newcastle upon Tyne UK

## Abstract

While common molecular anions show a strong propensity to undergo electron detachment upon UV excitation, this process often occurs in competition with molecular ion dissociation. The factors that affect the balance between these two major possible decay pathways have not been well understood to date. Laser photodissociation spectroscopy of the deprotonated forms of the UV filter molecules, Homosalate (HS) and Octyl Salicylate (OS), *i.e.* [HS − H]^−^ and [OS − H]^−^, was used to acquire gas-phase UV absorption spectra for [HS − H]^−^ and [OS − H]^−^*via* photodepletion from 3.0–5.8 eV. No photofragmentation (*i.e.* dissociation of the ionic molecular framework) was observed for either [HS − H]^−^ and [OS − H]^−^ following photoexcitation, revealing that electron loss entirely dominates the electronic decay pathways for these systems. High-level quantum chemical calculations were used to map out the excited states associated with [HS − H]^−^ and [OS − H]^−^, revealing that the minimum-energy crossing points (MECPs) between the S_1_ and S_0_ states are located in elevated regions of the potential energy surface, making internal conversion unlikely. These results are consistent with our experimental observation that electron detachment out-competes hot ground state molecular fragmentation. More generally, our results reveal that the competition between molecular dissociation and electron detachment following anion photoexcitation can be determined by the magnitude of the energy gap between the excitation energy and the MECPs, rather than being a simple function of whether the excitation energy lies above the anion's vertical detachment energy.

## Abstract 2

Laser photodissociation of deprotonated salicylates reveals that electron detachment outcompetes molecular dissociation following photoexcitation due to the locations of the minimum energy crossing points on the excited state surfaces.

## Graphical abstract

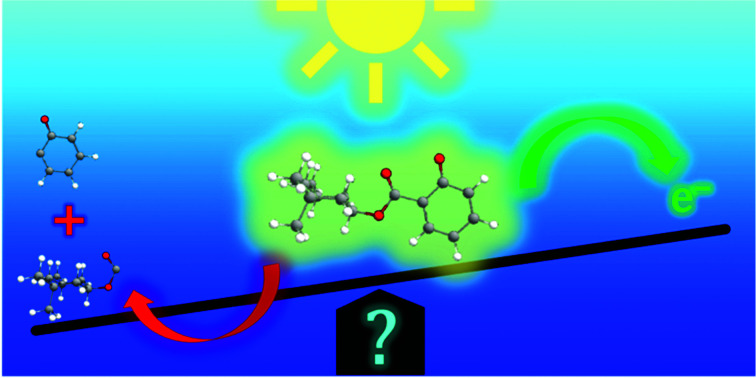

## 1. Introduction

The intrinsic photophysics of organic sunscreen molecules has been studied intensively over recent years *via* advanced laser spectroscopy and photochemistry experiments which aim to provide a more detailed picture of how sunscreens perform at a molecular level.^1,2^ One example of such studies is the application of laser interfaced mass spectrometry (LIMS) to probe how the behavior of protonated and deprotonated sunscreens differ, and hence to probe the effect of pH on sunscreens at the molecular level.^1^ These studies have revealed that protonation and deprotonation can alter the UV absorption profile of a sunscreen, as well as differentially affect the available decay pathways.^3,4^ Indeed, we have found that protonation/deprotonation can remove the ability of certain sunscreens to act effectively by perturbing the excited state decay pathways, rendering the molecules photo-unstable and prone to molecular dissociation.^3,4^

**Scheme 1:**
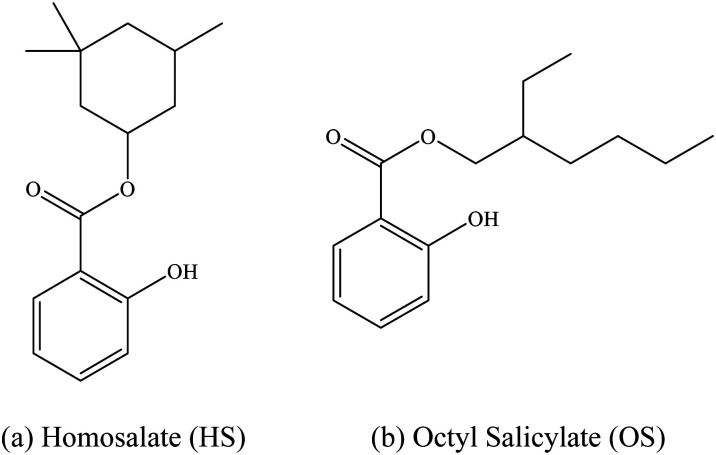
Schematic diagram of (a) homosalate (HS) and (b) octyl salicylate (OS).

In this work, we extend our previous LIMS studies of deprotonated UV filters to characterize the intrinsic UV absorption profile and photodegradation pathways of deprotonated homosalate (HS; Scheme 1) and octyl salicylate (OS; Scheme 1), *i.e.* [HS − H]^−^ and [OS − H]^−^, respectively. Salicylates such as HS and OS are relatively weak UV absorbers but are extremely commonly found in commercially available sunscreens and cosmetic skin care products. They are generally employed in these products to augment SPF (Sun Protection Factor) and enhance the water resistance of formulations, and function as excellent solubilizers for crystalline chemical UV filters like oxybenzone and avobenzone.^5,6^

The experiments we perform are supported by quantum chemical calculations to provide direct physical insight into how the photofragment identity maps the non-radiative relaxation channels. We are particularly interested here in investigating the extent to which photodissociation competes with electron loss following UV excitation. Although electron detachment is frequently the dominant decay pathway following UVA/UVB photoexcitation for isolated (*i.e.* gaseous) anions, branching into ionic molecular photodissociation channels can also be significant.^3,4,7^ To provide two examples from the UV filter molecules we have studied, deprotonated oxybenzone decays predominantly by electron detachment following UV photoexcitation whereas deprotonated 4-benzophenone photodecays with considerably higher yields of molecular photofragments.^3,7^ With the aid of the quantum chemical calculations performed as part of this work, we aim to obtain a better understanding of the factors that control electron detachment *versus* molecular dissociation branching. This is potentially an important consideration in organic sunscreen molecule design, since an enhanced propensity for electron detachment is associated with production of reactive free electrons which could be harmful in a biological context.

HS and OS are *ortho*-disubstituted molecules that possess a spatial arrangement allowing the formation of an intramolecular hydrogen bond which can facilitate excited-state intramolecular proton transfer (ESIPT) upon photoexcitation.^8,9^ In salicylates, ESIPT develops between the hydroxyl hydrogen and the carbonyl oxygen, and has been observed, in the case of methyl salicylate, to be maintained in the gas and solution phase.^10^ As seen previously in the case of the sunscreen oxybenzone,^3,11^ ESIPT is understood to facilitate the fast and efficient non-radiative decay to the electronic ground state, with excess UV energy being dissipated harmlessly as heat.^12–17^ This intramolecular hydrogen bond effectively lowers the excited-state energy, and accounts for its UV-B absorbance.^8,18^ Deprotonation of a salicylate molecule at the hydroxyl hydrogen therefore has the potential to strongly perturb its ability to undergo ESIPT, and in turn, lower their capacity to absorb in the UV-B, and perform effectively within a bulk sunscreen formulation.^1^ For that reason, it is important to examine the influence of protonation state on the intrinsic photochemical behaviors of HS and OS.

## 2. Experimental methods

### General

Homosalate (HS; 3,3,5-trimethylcyclohexyl salicylate) was purchased from Fluorochem Ltd (Glossop, UK) and octyl salicylate (OS; 2-ethylhexyl salicylate) from Tokyo Chemical Industry UK Ltd. (Oxford, UK), with both used as received. HPLC-grade EtOH was purchased from Fisher Scientific, Inc. (Pittsburgh, PA, USA), again used as received.

### Laser-interfaced mass spectrometry

Gas-phase UV photodissociation experiments were conducted in an AmaZon SL dual funnel electrospray ionization quadrupole ion trap (ESI-QIT) mass spectrometer (Bruker Daltonics Inc., Billerica, MA, USA), which was modified to allow for laser-interfaced mass spectrometry, as described previously.^19^ Solutions of HS (1.0 × 10^−4^ mol dm^−3^) in EtOH and subsequently OS (1.0 × 10^−4^ mol dm^−3^) in EtOH were introduced to the mass spectrometer *via* ESI, using typical instrumental parameters: nebulizing gas pressure of 10.0 psi, an injection rate of 0.33 mL h^−1^, a drying gas flow rate of 8.0 L min^−1^, and run in the negative ion mode at a capillary temperature of 160 °C to form their deprotonated species. Small quantities of dilute ammonia–water solutions (20 μL) were added to the solutions of HS and OS in EtOH prior to electrospray to enhance production of the deprotonated ions.

[HS − H]^−^ and [OS − H]^−^ were mass selected and isolated in the ion trap prior to UV laser irradiation. UV-Vis photons were produced by a 10 Hz Nd:YAG (Surelite™, Amplitude Laser Group, San Jose, CA, USA) pumped OPO laser (Horizon™, Amplitude Laser Group), giving ∼0.3 mJ across the range 400–216 nm (3.10–5.74 eV). Laser step sizes of 2 nm were used throughout. The laser beam was focused as described previously.^19^ Photofragmentation experiments were conducted with a set ion accumulation time of 10 ms and a corresponding fragmentation time of 100 ms, allowing for each mass-selected ion packet to interact with one laser pulse, and minimize the likelihood of multiphoton events. Note that photoproducts are not detected until the end of the 100 ms window, allowing us to identify photoproducts at the end of any decay processes. When fluorescence is negligible, the UV-excited gaseous ion will fragment upon excited-state relaxation, yielding an action absorption spectrum by photodepletion.^19–21^

### Analysis

Photodepletion (PD) of [HS − H]^−^ and [OS − H]^−^ was measured as a function of scanned wavelength, with the photofragment production (PF) recorded simultaneously at each corresponding wavelength.1a
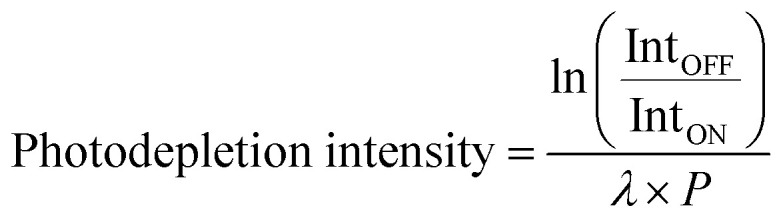
1b
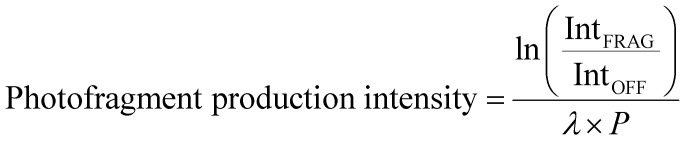


As shown in eqn (1a) and (1b), Int_OFF_ and Int_ON_ represent the parent ion intensities with laser off and on, respectively; Int_FRAG_ is the photofragment intensity with the laser on; *λ* is the excitation wavelength (nm); and *P* is the tunable laser pulse energy (mJ). The photodepletion intensities were taken from an average of three repeat runs at each wavelength within the range studied. We note that photofragment ions with *m*/*z* < 50 are not detectable within our mass spectrometer since low masses fall outside of the mass window of the ion trap.

### Collision-induced dissociation

Low-energy collision-induced dissociation (CID) was employed to determine the ground state thermal fragmentation productions of [HS − H]^−^ and [OS − H]^−^. This technique was performed on isolated ions by applying an excitation AC voltage to the end caps of the trap to induce collisions of the trapped anions with the He buffer gas, as described in detail previously.^22–24^ Excitation is similar to thermal excitation in a hot, low-pressure gas, so that when the high-energy tail of the ion energy envelope reaches activation energy for the lowest-energy decomposition pathway, decomposition begins to occur, and fragment ions are produced. The fragment ions observed therefore characterize the low-energy potential energy surface (*i.e.*, the surface with the lowest barrier to fragmentation).^22–24^

### Computational details

All accompanying resolution-of-identity (RI) Møller–Plesset perturbation theory/second-order algebraic diagrammatic construction [RI-MP2/ADC(2)] calculations were carried out using TURBOMOLE (v7.4.0).^25–27^ RI-MP2/ADC(2) calculations^28–31^ employed the CC2 routines implemented in TURBOMOLE^31–35^ and used the frozen-core approximation; the 19 and 18 lowest-energy core orbitals of [HS − H]^−^ and [OS − H]^−^, respectively, were frozen in all RI-MP2/ADC(2) calculations. A tightened SCF convergence criterion of 1.0 × 10^−8^ a.u. was used in all calculations; convergence criteria of 1.0 × 10^−6^ and 3.0 × 10^−4^ a.u. were used for the energy change and RMS gradient, respectively, in all geometry optimizations. The aug-cc-pVDZ basis set of Dunning *et al.*^36,37^ was used throughout and coupled with the correlated aug-cc-pVDZ/C auxiliary basis set. The proper convergence of all geometry optimizations to real minima was verified *via* vibrational frequency inspection.

Minimum-energy crossing points (MECP) between the first electronically-excited singlet (S_1_) and ground electronic (S_0_) states of [HS − H]^−^ and [OS − H]^−^ were located *via* a home-built external objective function (*F*_*ij*_) optimizer following the approach of Martinez *et al.*;^38^ convergence was indicated when the change in the value of the objective function, Δ*F*_*ij*_, was below 1.0 × 10^−6^ a.u., provided that the convergence criterion of 3.0 × 10^−4^ for the RMS gradient was simultaneously satisfied. Initial values of *σ* and *α* were set to 3.50 and 0.02, respectively. The quality of reference wavefunction was assessed *via D*_1_ diagnostic inspection and reporting throughout. Ref. 39 and 40 provide further background on the importance of MECPs in the mechanism of internal conversion.^39,40^

## 3. Results and discussion

### 3.1 Gas-phase UV absorption spectra of deprotonated homosalate and octyl salicylate

**Fig. 1:**
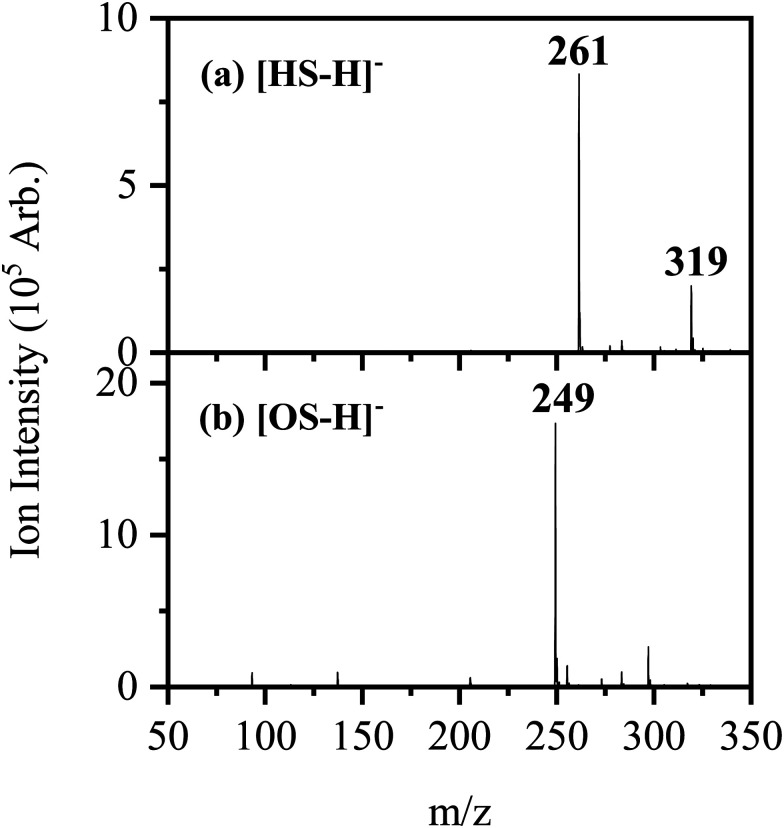
Negative ion electrospray ionization mass spectra of (a) [HS − H]^−^ (*m*/*z* 261) and (b) [OS − H]^−^ (*m*/*z* 249), respectively.

Fig. 1 shows the electrospray ionization mass spectra obtained when solutions of (a) HS in EtOH and (b) OS in EtOH are sprayed in negative ion mode. Both the [HS − H]^−^ and [OS − H]^−^ ions can be produced as intense molecular anions, and fragmentation into lower *m*/*z* ions appears limited, indicating that the anions are intrinsically stable upon electrospray.

**Fig. 2:**
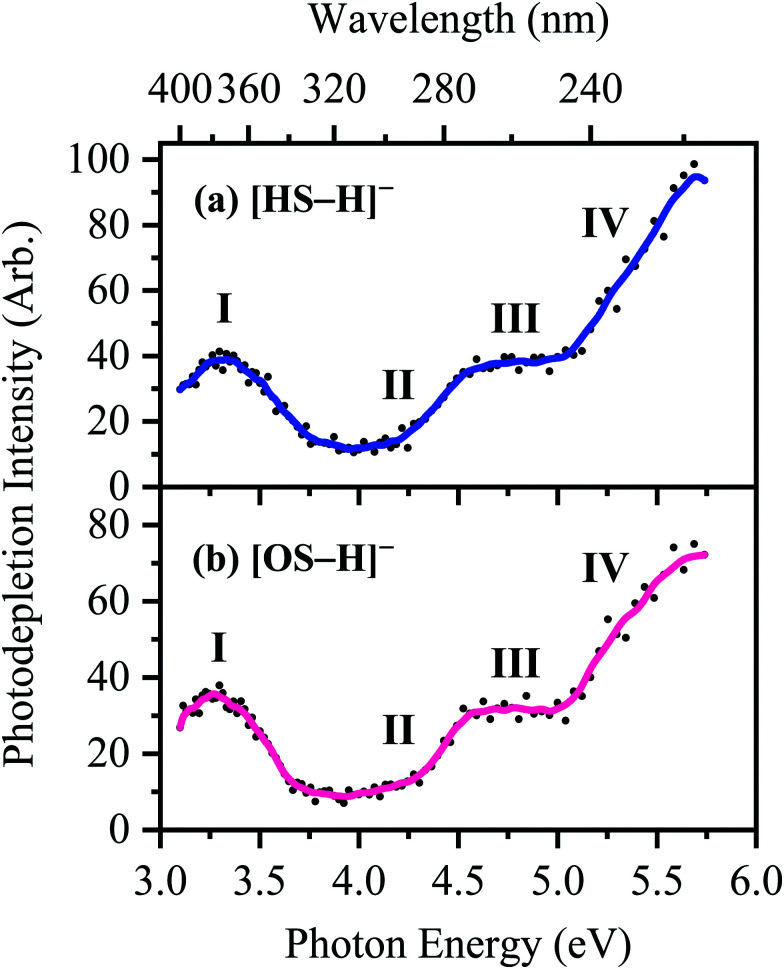
Gas-phase UV absorption (photodepletion) spectra of (a) [HS − H]^−^ and (b) [OS − H]^−^, respectively. The solid line is a five-point adjacent average of the data points.

The photodepletion (gas-phase absorption) spectrum of mass-selected [HS − H]^−^ across the 3.10–5.74 eV (400–216 nm) range is shown in Fig. 2a. Mass selection is a key advantage of the experimental approach we employ here as it allows us to directly probe the intrinsic properties of the deprotonated systems. The gaseous absorption spectrum of [HS − H]^−^ displays strong absorption in the UV-A region (with an onset below the lowest measured photon energy of 3.1 eV) through a band I which then reduces in intensity through the higher UV-B region (3.6–4.2 eV). Absorption again increases between 4.2–4.5 eV (II), and subsequently plateaus through the high UV-C wavelength region (III). Band IV therefore marks the onset of an intense broad photodepletion region which extends into the lower UV-C.

The photodepletion spectrum of [OS − H]^−^ (*m*/*z* 249), over the range 3.10–5.74 eV, is displayed in Fig. 2b. The gas-phase absorbance profile can be seen to be extremely similar to that of the spectra of [HS − H]^−^ (Fig. 2a), reflecting the similarity of the chromophores for both molecules.

An important aspect of our gas-phase LIMS experiment is that it allows us to identify the ionic photofragments produced following UV excitation of an isolated precursor ion.^3,4,7,19^ However, in this case, no photofragments were observed for either [HS − H]^−^ or [OS − H]^−^ across the entire UV region scanned. (We note that only fragments with approximately *m*/*z* > 50 can be detected in our ion-trap mass spectrometer.) This situation is highly unusual, with [HS − H]^−^ or [OS − H]^−^ representing the first two molecules we have studied by LIMS which do not produce ionic photofragments following photoexcitation.^1,3,4,41^ To further explore the possible ionic breakdown pathways for these ions, we proceeded to study them using collision-induced dissociation (CID).

### 3.2 Thermal fragmentation of deprotonated homosalate and octyl salicylate

**Fig. 3:**
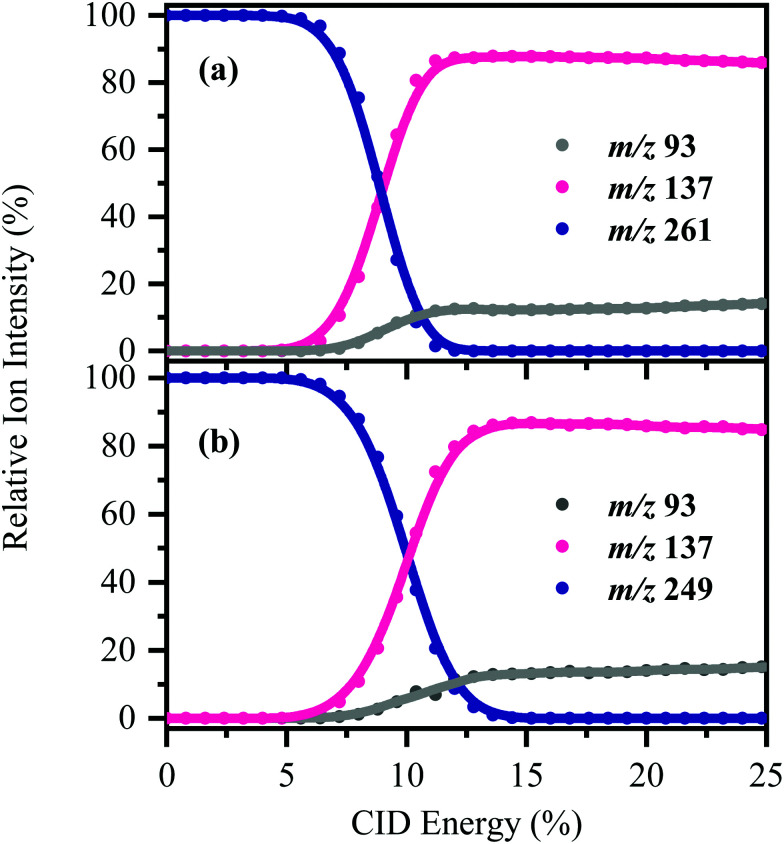
CID fragmentation curves of (a) [HS − H]^−^ and (b) [OS − H]^−^ between 0–25% CID energy. Onset plots for production of the associated fragment ions (*m*/*z* 93 and 137) are also shown. The curved lines are a three-point adjacent average of the data points.

[HS − H]^−^ and [OS − H]^−^ were subjected to low-energy CID to determine the pathways associated with fragmentation of the ground-electronic state. Fig. 3 displays the low-energy CID fragmentation curves for (a) [HS − H]^−^ and (b) [OS − H]^−^, respectively. CID results in production of the *m*/*z* 137 ion as the dominant fragment for both [HS − H]^−^ and [OS − H]^−^, with the *m*/*z* 93 fragment being produced at relatively lower intensities (<15%). Possible assignments of the *m*/*z* 137 and 93 fragments observed are given in Table 1, with the *m*/*z* 137 fragment likely resulting from loss of the alkyl side chain, with the *m*/*z* 93 fragment resulting from additional loss of CO_2_.

**Table 1:** Proposed structures for the ionic fragments of [HS − H]^−^ (*m*/*z* 261) and [OS − H]^−^ (*m*/*z* 249) produced upon collision-induced dissociation (CID)

Ionic fragment mass (*m*/*z*)	Proposed structure of ionic fragment	CID fragment of [HS − H]^−^ and [OS − H]^−^
137	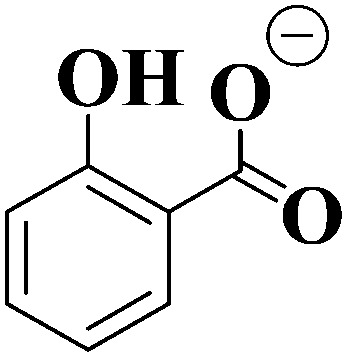	✓ (strong)
93	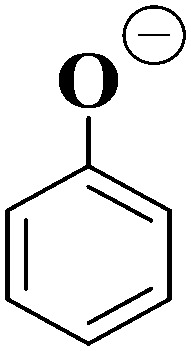	✓ (weak)

The fact that stable fragments can be observed from [HS − H]^−^ and [OS − H]^−^ following CID illustrates that these systems can undergo molecular dissociation with production of stable ionic fragments, *i.e.* electron detachment is not the only potential breakdown pathway available to these anions. We note that in other organic sunscreen molecules we have studied,^3,4,7,41^ the ions associated with ground electronic state fragmentation (*i.e.* the CID fragments) have been observed following photoexcitation. This behavior has been traced to rapid internal conversion occurring on the excited state surface, followed by molecular dissociation of the system on the hot electronic ground state.^7^ To better understand why this does not occur for [HS − H]^−^ and [OS − H]^−^, we turn to quantum chemical calculations.

### 3.3 Quantum chemical calculations and interpretation

High-level *ab initio* wavefunction theory calculations at the [RI-MP2/ADC(2)]/aug-cc-pVDZ level were performed to further probe the photodecay of [HS − H]^−^ and [OS − H]^−^. These calculations were performed under a ‘single-conformer’ approximation for each of [HS − H]^−^ and [OS − H]^−^ in order to make the most efficient use of the available computational resources. This approximation is justified by the following arguments: (a) the conformation(s) of the alkyl ester group does not appear to have an appreciable effect on the experimental observables (*i.e.*, the UV/vis absorption/photodepletion spectra of the two systems are similar), particularly given the limited spectral resolution, and (b) the number of possible alkyl ester group conformations in [OS − H]^−^ (by way of an example) is too large to treat effectively at the [RI-MP2/ADC(2)]/aug-cc-pVDZ level, even after reduction *via* screening. In each case, the conformer considered is the lowest-energy ground-state conformer locatable at the [RI-MP2/ADC(2)]/aug-cc-pVDZ level.

The *C*_1_-symmetric S_0_ minimum-energy geometries of [HS − H]^−^ and [OS − H]^−^ were located at the [RI-MP2/ADC(2)]/aug-cc-pVDZ level (Tables S1 and S2, ESI†), and key parameters relating to electron detachment [vertical detachment energies (VDEs) and vertical dipole moments (VDMs)] at these geometries are tabulated in Table 2. The *C*_1_-symmetric *D*_0_ minimum-energy geometries of the neutral counterparts, [HS − H]˙ and [OS − H]˙, were also located at the [RI-MP2/ADC(2)]/aug-cc-pVDZ level (Tables S3 and S4, ESI†), and these have been used to determine supplementary adiabatic electron detachment energies (ADEs) which are, additionally, tabulated in Table 2. The greater difference between the VDE and ADE in [OS − H]^−^ (*ca.* 0.4 eV) compared to [HS − H]^−^ (<0.1 eV) is reflective of the greater geometric reorganization in the former (a consequence of the greater flexibility of the alkyl ester group to respond to the charge state) compared to the latter; [OS − H]^−^ and [OS − H]˙ are separated in space by 18.3 Å Da^−1/2^, while [HS − H]^−^ and [HS − H]˙ are only separated in space by 2.6 Å Da^−1/2^.

**Table 2:** Summary of vertical and adiabatic detachment energies (VDEs/ADEs)^a^^b^ in eV and vertical dipole moments (VDMs)^c^ in Debye for [HS − H]^−^ and [OS − H]^−^ as evaluated at the [RI-MP2/ADC(2)]/aug-cc-pVDZ level

System	VDE^a^/eV	ADE^b^/eV	VDM^c^/D
[HS − H]^−^	3.90	3.88	2.33
[OS − H]^−^	4.34	3.90	5.97

a VDE = *E*_neutral_ – *E*_anion_ (at the optimized anion geometry).

b ADE = *E*_neutral_ – *E*_anion_ (at the optimized neutral and anion geometries, respectively).

c VDM = *μ*_neutral_ (at the optimized anion geometry). Vertical dipole moments are important quantities in assessing whether a dipole-bound excited state is possible for these anions. See ref. 43 for more details.^42^

**Fig. 4:**
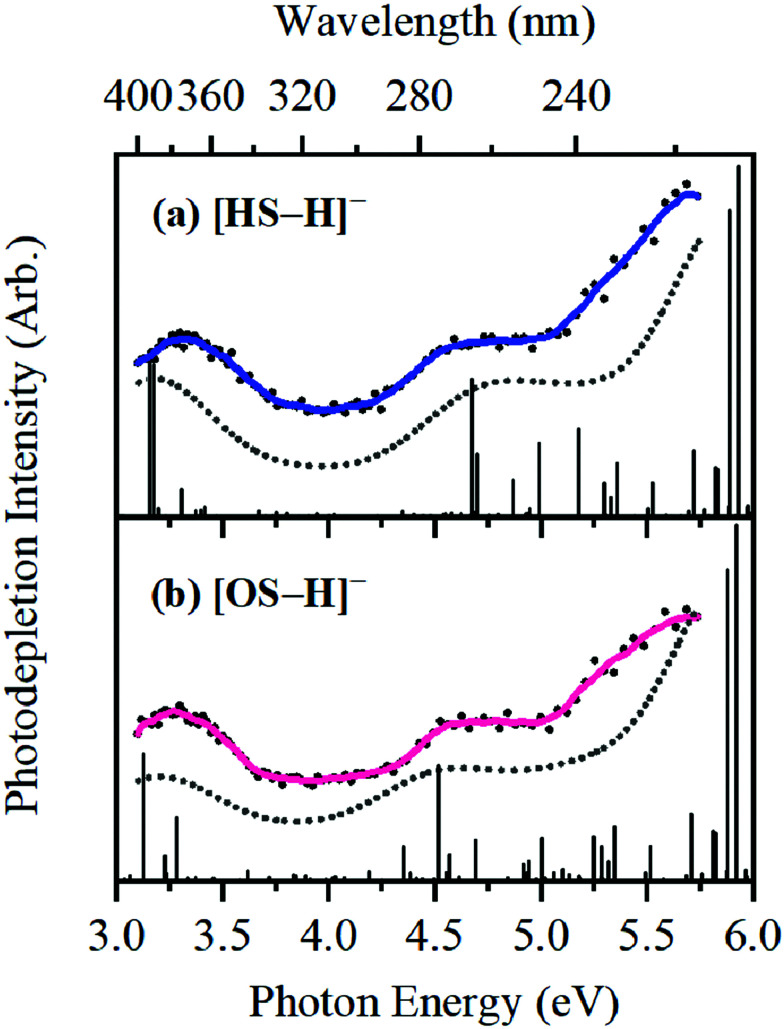
Gas-phase experimental photodepletion spectra (solid colored line) *vs.* theoretical UV absorption spectra (dotted black line) calculated at the [RI-MP2/ADC(2)]/aug-cc-pVDZ level for (a) [HS − H]^−^ and (b) [OS − H]^−^, respectively; theoretical UV absorption spectra were phenomenologically broadened with Lorentzian functions (FWHM = 0.8 eV). The vertical lines represent the calculated vertical excitation energies.

Bands II–IV in [HS − H]^−^ and [OS − H]^−^ are embedded in the electron detachment continuum (*i.e.*, they are located above the VDEs), and it is reasonable to assume that electronic excitation at these bands produces excited states that decay primarily *via* electron detachment, *i.e.*, without photofragmentation, while Band I is located below (yet in proximity to) the electron detachment threshold; consequently, other decay mechanisms, *e.g.*, S_0_ ← S_1_ internal conversion (IC) or fluorescence/phosphorescence, cannot be ruled out in such a straightforward sense. However, the calculated gas-phase absorption spectra of [HS − H]^−^ and [OS − H]^−^ are in good agreement with the photodepletion spectra (Fig. 4), which demonstrates that luminescence processes are not competitive for either of these systems. (If they were, there would be a mismatch between the absorption spectrum and photodepletion since luminescence decay is not evident as photodepletion of the precursor ion.)^20^

**Fig. 5:**
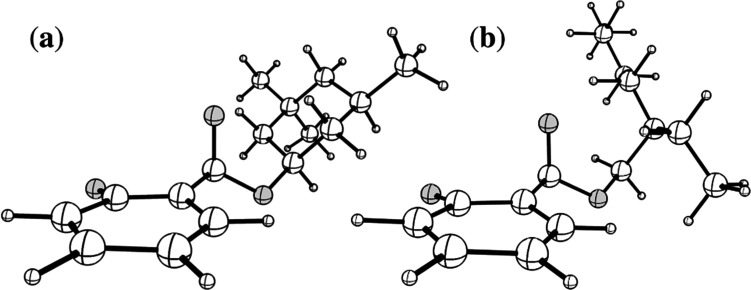
Illustrations of the geometries of the S_0_ ← S_1_ MECP of (a) [HS − H]^−^ and (b) [OS − H]^−^. Atom labels for both molecules are given in Fig. S1 of the ESI.†

A naïve search for S_0_ ← S_1_ MECP in [HS − H]^−^ and [OS − H]^−^, starting from the Franck–Condon point, reveals a false S_0_ ← S_1_ IC channel along the C_2_=O_10_ (carbonyl) stretching coordinate; recent work by Marsili, Prlj, and Curchod^43^ has demonstrated these artificial S_0_ ← S_1_ MECP to be the product of systematic weaknesses of RI-MP2/ADC(2) for this class of systems. The real S_0_ ← S_1_ MECP in [HS − H]^−^ and [OS − H]^−^ are quasi-ethylenic, *i.e.*, they are associated with twisting about a bond and pyramidalisation of one of the bond termini. These S_0_ ← S_1_ MECP were located at the [RI-MP2/ADC(2)]/aug-cc-pVDZ level (Tables S5 and S6, ESI†); their geometries are illustrated in Fig. 5, and key geometric parameters are tabulated in Table 3.

**Table 3:** Summary of relative energies (Δ*E*)^a^ in eV, distances (*d*)^a^ in Å Da^−1/2^, and key geometric parameters^b^^,c^ for the S_0_ ← S_1_ MECP in [HS − H]^−^ and [OS − H]^−^ as evaluated at the [RI-MP2/ADC(2)]/aug-cc-pVDZ level

System	Δ*E*^a^/eV	*d* a/Å Da^−1/2^	*ϕ*C_2_–C_1_–C_7_–O_8_/°	*ϕ*C_2_–C_1_–C_7_–O_9_/°	*θ*_pyr_.^b^	*θ*_w_.^c^
[HS − H]^−^	+0.61	16.6	88.2	32.6	0.091	8.2
[OS − H]^−^	+0.94	21.9	82.8	26.0	0.098	8.6

a Relative to the S_1_-state Franck–Condon point.

b *θ*_pyr_. is a pyramidalisation index quantifying the extent of the pyramidalisation at the C_7_ site; it is defined here as 1.0 − ((*α* + *β* + *γ*)/360.0), where *α*, *β*, and *γ* are the angles around the C_7_ site.

c *θ*_w_. is an index quantifying the out-of-plane displacement of O_10_; it is defined here as the angle between the C_2_–O_10_ vector and the plane containing the aromatic ring (C_1_–C_2_–C_3_–C_4_–C_5_–C_6_).

**Fig. 6:**
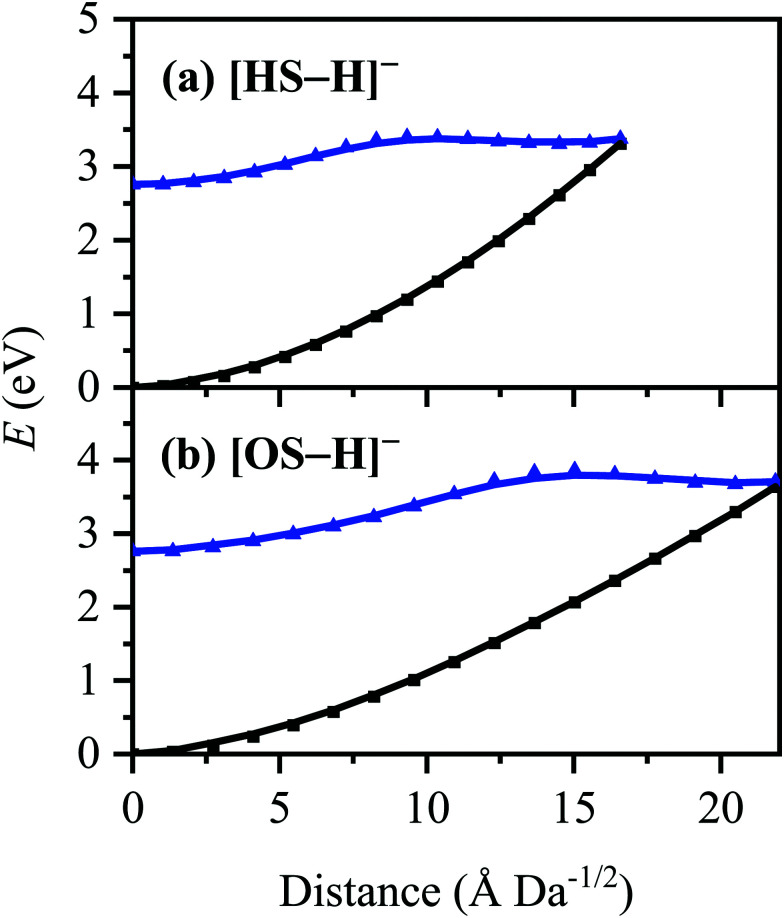
Energies of the S_0_ (black) and S_1_ (blue) states between the S_0_ minimum-energy geometries/Franck–Condon points and the S_0_ ← S_1_ MECP of (a) [HS − H]^−^ and (b) [OS − H]^−^. Points were generated *via* linear interpolation of internal coordinates (LIIC). Energies were evaluated at the [RI-MP2/ADC(2)]/aug-cc-pVDZ level.

The S_0_ ← S_1_ MECP in [HS − H]^−^ and [OS − H]^−^ are located 0.61 and 0.94 eV above the S_1_-state Franck–Condon point (Table 3), respectively, and 0.23 and 0.57 eV above the peak of Band I, *i.e.*, they are not readily accessible and are, to an extent, barrier-activated. To map the S_0_ ← S_1_ IC channel, potential energy surfaces have been constructed between the Franck–Condon points and S_0_ ← S_1_ MECP of [HS − H]^−^ and [OS − H]^−^*via* linear interpolation of internal coordinates (LIIC). Independent single-point energy calculations have been carried out at each one of 15 interpolated geometries, with the calculated potential energy surfaces for [HS − H]^−^ and [OS − H]^−^ presented in Fig. 6a and b, respectively.

Although barrier heights determined *via* LIIC represent an upper bound to the true barrier height at the applied level of theory [*i.e.*, a reduction in the barrier height might be expected following a minimum-energy pathway (MEP) scan, for example], it is nonetheless apparent that these S_0_ ← S_1_ MECP are located in elevated regions of the potential energy surface and are not likely to be accessed readily without a surplus of excitation energy that would place the excitation into the electron detachment continuum. This is qualitatively consistent with the recent time-resolved laser spectroscopic study on HS carried out by Holt and Stavros *et al.*;^44^ the authors recorded decays associated with two time constants – one ultrafast process corresponding to excited-state intramolecular proton transfer (ESIPT; not observed here in the deprotonated system) and a much longer process corresponding to excited-state decay.^45^ The time constant for the latter process was found to have an associated energy dependence, consistent with a barrier being presented to S_0_ ← S_1_ IC.^45,46^ The authors did not locate the S_0_ ← S_1_ MECP for HS, but we expect it closely resembles the one located here for [HS − H]^−^.

The consequence of these calculations is that excited states formed *via* excitation at Band I are unlikely to decay *via* the identified S_0_ ← S_1_ IC channel; the generation of molecular fragments on the hot ground state (observed *via* CID; Fig. 3) is not observed post-photoexcitation in our LIMS experiments because the systems do not return non-radiatively to the hot ground state *via* IC. This means that the only channels available to these ions following photoexcitation at Band I are either fluorescence/phosphorescence, or electron detachment. As discussed earlier in this section, the excellent agreement between the calculation absorption spectrum (Fig. 4) and the experimental photodepletion spectra means that luminescence processes are not significant, allowing us to conclude that electron detachment is dominant.

Finally, we note that the S_1_ states of both [HS − H]^−^ and [OS − H]^−^ occur at energies below the respective calculated ADEs, so that electron detachment would not be predicted to be an energetically decay pathway. We have, however unambiguously observed such behavior in several other systems, with electron detachment being observed experimentally significantly below the expected electron detachment energies.^47,48^ This is likely due to the considerable residual internal energy of the anions in our experiment, which are produced at ambient temperatures through the electrospray ionization source.

## 4. Concluding remarks

The intrinsic photophysics and photochemistry of organic UV filtering molecules has been explored over recent years to improve our fundamental understanding of their efficiency and photostability,^49,50^ as well as their potential as environmental pollutants.^51–53^ Since HS and OS are often found at high concentrations (∼5–15%) within commercial sunscreens and cosmetic products, a fundamental understanding of their intrinsic photostability is important.^6^ In the work presented above, we demonstrate the utility of LIMS photodissociation mass spectrometry to probe the intrinsic UV absorption profile and photodegradation pathway(s) of the two structurally-similar organic UV-B filters, HS and OS in their deprotonated forms. We found that in the gas-phase, both [HS − H]^−^ and [OS − H]^−^ have similar UV absorption profiles, and decay following photoexcitation as isolated molecules solely *via* electron detachment, without production of ionic molecular fragments. As detailed above, these observations are consistent with the excited state anions being unable to readily access the potential energy crossing points to undergo ultrafast internal conversion. This leads us to conclude that the isolated, deprotonated forms of HS and OS are not behaving as efficient sunscreen molecules, where absorption of a UV photon is followed by internal conversion back to a hot electronic ground state. We note that this behavior may change in the condensed phase environment of a commercial sunscreen mixture. Electron detachment energies are typically reduced upon solvation of an anion by a water molecule, but the extent to which such stabilization will occur in an oily (less-polar) sunscreen is unclear. Our work suggests that experiments to explore the extent to which electron detachment occurs in condensed mixtures of HS and OS are merited.

Our results for [HS − H]^−^ contrast to those observed by Holt *et al.* for neutral HS, where time-resolved ion-yield spectroscopy showed that UV photoexcited gaseous HS undergoes ultrafast decay with a time constant of >170 fs as the dominant excited-state decay pathway.^44^ Calculations performed to support these measurements suggest that photoexcitation is followed by excited state intramolecular proton transfer (ESIPT), where the enol form of HS is converted to the keto tautomer, followed by rapid non-radiative dissipation of the excess energy. This represents prototypical behavior for a well-behaved organic sunscreen molecule, involving the conversion of UV photons to thermal energy. Our results for [HS − H]^−^ indicate that the ultrafast decay pathway available to neutral HS is lost upon deprotonation, due to removal of the most acidic proton occurring at the enol group. Hence, ESIPT can no longer occur. This evolution from a functioning sunscreen molecule to one which displays poor sunscreen action upon deprotonation mirrors behavior we have seen previously in oxybenzone,^3^ another common sunscreen molecule.

The pathways available following photoexcitation of an anionic molecule or cluster are complex.^54,55^ Below the electron detachment energy, photoexcitation can only access valence excited states, which can decay radiatively or through dissociation into an anionic and neutral fragment pair. Above the detachment energy, electronic excited states are coupled to the electron detachment continuum,^56^ and can thus be considered autodetaching resonances. Electron detachment can either occur directly from the excited state or following excited state decay back to the ground electronic state and ensuing thermionic emission.^57–60^ Similar pathways are possible for molecular photodissociation,^4,7^ with formation of two neutral photofragments following electron detachment being an additional possibility.^61,62^ The high-level calculations performed as part of this work reveal that the competition between molecular dissociation and electron detachment following anion photoexcitation of the deprotonated UV filters [HS − H]^−^ and [OS − H]^−^ is determined by the magnitude of the energy gap between the excitation energy and the MECPs, rather than being a simple function of whether the excitation energy lies above the anion's vertical detachment energy. Our current understanding is sparse of how the potential energy surfaces will change in the oily, condensed-phase environment of a sunscreen mixture.^63^ Nonetheless, the issue is potentially important given the harmful nature of free electrons in a biological context, and merits further investigation for the systems studied here.

## Conflicts of interest

There are no conflicts to declare.

## Supplementary Material

CP-024-D2CP01612E-s001
