# Supplementary material for: Photostability of the deprotonated forms of the UV filters homosalate and octyl salicylate: molecular dissociation versus electron detachment following UV excitation
Source: Phys Chem Chem Phys. 2022 Jun 27;24(28):17068–76. doi: 10.1039/d2cp01612e (PMC9301628; doi:10.1039/d2cp01612e)
Supplement: CP-024-D2CP01612E-s001 [file CP-024-D2CP01612E-s001.pdf]

Electronic Supplementary Material (ESI) for Physical Chemistry Chemical Physics.
This journal is © the Owner Societies 2022

SUPPORTING INFORMATION

Photostability of the Deprotonated Forms of the UV Filters Homosalate and Octyl Salicylate: Does Dissociation Compete with Electron Detachment Following UV Excitation?

Natalie G. K. Wong, Conor D. Rankine, Cate S. Anstöter and Caroline E. H. Dessent*

Department of Chemistry, University of York, Heslington, YO10 5DD, U. K.

* E-mail: caroline.dessent@york.ac.uk

Supporting Information:

S1. Photodepletion Laser Power Dependence Measurements

S2. Optimized Cartesian Coordinate Tables

S3. Schematic Structure of the $[HS-H]^-$ and $[OS-H]^-$ Chromophore

S1. Laser Power Dependence Measurements

Laser power measurements were conducted on $[HS-H]^-$ and $[OS-H]^-$ at their respective absorption maxima of 3.31 and 5.69 eV. The plots displayed in Figure S1 are that of the parent ion photodepletion intensities ($Int_{OFF} - Int_{ON}$) at such photon energies. Following standard protocol, such data has been plotted and fit to a power function. The resultant slope is thereby proportional to the number of absorbed photons implicated in this experiment. Multiphoton events *via* instantaneous absorption of multiple photons in the Franck-Condon region are negligible as the laser beam is only softly focused through the ion trap region. The slopes at all four photon energies measure at less than 1, confirming that photodepletion of $[HS-H]^-$ and $[OS-H]^-$ at 0.3 mJ are evidently not multiphoton in nature.

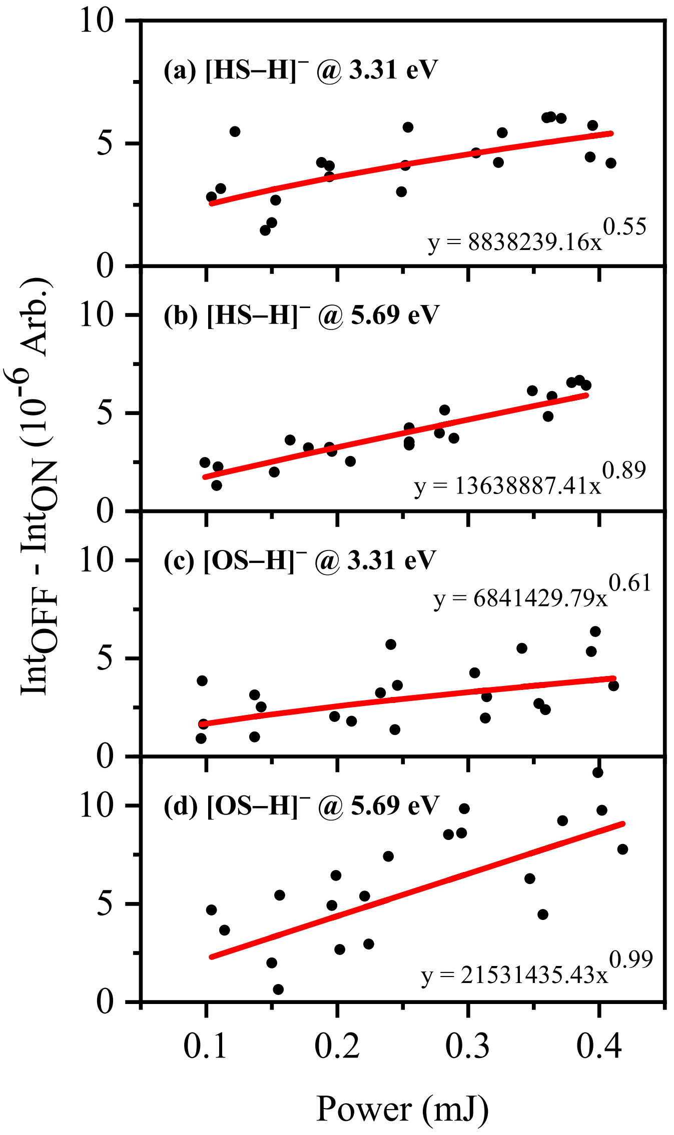

Figure S1. Power dependence measurements for [HS-H]$^-$ at two absorption maxima of **(a)** 3.31 eV and **(b)** 5.69 eV and [OS-H]$^-$ at two absorption maxima of **(c)** 3.31 eV and **(d)** 5.69 eV.

S2. Optimized Cartesian Coordinate Tables

Table S1. Optimized Cartesian coordinates in Å and ground-state RI-MP2 energies, $E_{\text{RI-MP2}}$, in atomic units (a.u.) for the minimum-energy geometry of $[\text{HS-H}]^-$ at the RI-MP2/*aug*-cc-pVDZ level.

$E_{\text{RI-MP2}} = -845.7314898567$			
C	−1.970469	−2.648821	−0.389568
C	−2.426585	−3.717271	0.514986
C	−3.368625	−4.649605	−0.103317
C	−3.804244	−4.552660	−1.421412
C	−3.338374	−3.504621	−2.261655
C	−2.435979	−2.578230	−1.733613
C	−1.021767	−1.637627	0.095399
O	−0.520944	−1.538220	1.211892
O	−0.702086	−0.704628	−0.916454
C	0.218472	0.352575	−0.578746
C	−0.325786	1.311352	0.487810
C	0.603867	2.526103	0.704728
C	2.033187	2.019233	0.992213
C	2.581332	1.057283	−0.074520
C	1.626251	−0.138324	−0.223013
O	−2.070200	−3.865206	1.724938
C	0.596286	3.464070	−0.515886
C	0.101910	3.319407	1.921023
C	3.994165	0.587437	0.292480
H	−3.728190	−5.458143	0.544863

H	−4.514162	−5.293976	−1.812361
H	−3.676545	−3.421372	−3.299610
H	−2.068841	−1.766760	−2.367443
H	0.288956	0.898757	−1.537317
H	−0.430572	0.757412	1.433610
H	−1.331692	1.656745	0.185094
H	0.947235	2.969002	−1.433710
H	−0.426629	3.833868	−0.703629
H	1.248264	4.336945	−0.332371
H	0.749461	4.193346	2.116168
H	−0.926045	3.684419	1.750959
H	0.093739	2.684061	2.822203
H	2.022918	1.479711	1.960335
H	2.712804	2.886853	1.110968
H	2.639552	1.592312	−1.042481
H	3.976277	0.047933	1.254885
H	4.394343	−0.098558	−0.472788
H	4.688591	1.440077	0.388722
H	1.589956	−0.703110	0.722180
H	1.980169	−0.821768	−1.015563

Table S2. Optimized Cartesian coordinates in Å and ground-state RI-MP2 energies, $E_{\text{RI-MP2}}$, in atomic units (a.u.) for the minimum-energy geometry of $[OS\text{-}H]^-$ at the RI-MP2/*aug*-cc-pVDZ level.

$E_{\text{RI-MP2}} = -807.7228143538$			
C	−0.488781	−1.236721	1.884044
C	−0.981849	−0.135940	2.715850
C	−0.601191	−0.254611	4.114936
C	0.194699	−1.289354	4.612190
C	0.688446	−2.308145	3.757393
C	0.349214	−2.255913	2.398813
C	−0.843560	−1.250794	0.455421
O	−1.894753	−0.890414	−0.058459
O	0.224192	−1.729801	−0.327500
C	0.153007	−1.361276	−1.717314
C	0.971160	−0.078575	−1.934565
C	0.372596	1.113038	−1.164599
C	−0.993497	1.602474	−1.659474
C	−1.634154	2.576384	−0.662964
C	−3.024095	3.036181	−1.117892
C	2.440155	−0.274163	−1.512964
C	3.146127	−1.461844	−2.180667
O	−1.644180	0.862358	2.275329
H	−0.965144	0.535024	4.783523
H	0.442044	−1.309160	5.681901
H	1.315455	−3.116686	4.146500
H	0.713146	−3.028737	1.712788

H	−0.897061	−1.227161	−2.017080
H	0.575826	−2.204646	−2.285826
H	0.938969	0.139696	−3.023759
H	1.093039	1.952785	−1.207955
H	0.290988	0.844413	−0.095326
H	−0.882161	2.080594	−2.653900
H	−1.673803	0.743638	−1.778279
H	−1.707820	2.069122	0.315678
H	−0.974048	3.455142	−0.532203
H	−2.982838	3.540791	−2.100302
H	−3.698802	2.168531	−1.208127
H	−3.473538	3.736071	−0.394132
H	2.987912	0.656505	−1.747857
H	2.467831	−0.395642	−0.416659
H	4.223845	−1.460253	−1.947910
H	2.734489	−2.420496	−1.829583
H	3.038137	−1.422413	−3.279073

Table S3. Optimized Cartesian coordinates in Å and ground-state RI-MP2 energies, $E_{\text{RI-MP2}}$, in atomic units (a.u.) for the minimum-energy geometry of [HS-H]$^{\bullet}$ at the RI-MP2/*aug*-cc-pVDZ level.

$E_{\text{RI-MP2}} = -845.6055186348$

C	−1.957017	−2.610520	−0.586997
C	−2.309721	−3.579262	0.352877
C	−3.269448	−4.550367	−0.000288
C	−3.764914	−4.518921	−1.257684
C	−3.416044	−3.579052	−2.220681
C	−2.523821	−2.647459	−1.852593
C	−1.033200	−1.599847	−0.077250
O	−0.649600	−1.597786	1.100543
O	−0.687710	−0.680165	−1.014038
C	0.240124	0.392507	−0.633205
C	−0.333509	1.313610	0.443529
C	0.599021	2.517304	0.712526
C	2.014662	1.989629	1.029999
C	2.591245	1.048633	−0.041527
C	1.627103	−0.133060	−0.263834
O	−1.762572	−3.645742	1.501686
C	0.623212	3.495656	−0.473195
C	0.064337	3.270781	1.941050
C	3.980952	0.541813	0.361484
H	−3.581965	−5.293478	0.733853
H	−4.499066	−5.290370	−1.522647
H	−3.850458	−3.607056	−3.218960

H	−2.216507	−1.867161	−2.557460
H	0.310842	0.941773	−1.584927
H	−0.459862	0.742088	1.375626
H	−1.329238	1.674027	0.125957
H	1.004596	3.044028	−1.401077
H	−0.393559	3.873963	−0.674511
H	1.266389	4.360103	−0.235352
H	0.709056	4.133794	2.180970
H	−0.956403	3.647458	1.756290
H	0.031710	2.608729	2.822234
H	1.975010	1.435850	1.988444
H	2.697710	2.847362	1.181768
H	2.693242	1.606933	−0.990836
H	3.921833	−0.022568	1.307557
H	4.399671	−0.126554	−0.408784
H	4.682081	1.380209	0.505961
H	1.561410	−0.726665	0.662393
H	2.000408	−0.790220	−1.068902

Table S4. Optimized Cartesian coordinates in Å and ground-state RI-MP2 energies, $E_{\text{RI-MP2}}$, in atomic units (a.u.) for the minimum-energy geometry of [OS-H]$^{\bullet}$ at the RI-MP2/*aug*-cc-pVDZ level.

$E_{\text{RI-MP2}} = -807.5631954953$			
C	−0.270937	−1.467071	2.038417
C	0.829165	−0.665919	2.629032
C	1.114231	−0.932844	4.053794
C	0.377310	−1.819886	4.774183
C	−0.690586	−2.519775	4.165892
C	−1.001393	−2.321174	2.800132
C	−0.608072	−1.298839	0.585037
O	−1.739685	−1.060981	0.182358
O	0.484382	−1.498792	−0.173390
C	0.354557	−1.193151	−1.599415
C	0.975652	0.177148	−1.872843
C	0.159706	1.314155	−1.234129
C	−1.245672	1.508083	−1.812263
C	−2.016141	2.623657	−1.096577
C	−3.425448	2.819707	−1.667272
C	2.438879	0.244076	−1.394294
C	3.330248	−0.889461	−1.917575
O	1.432884	0.185486	2.003693
H	1.932934	−0.356688	4.490399
H	0.597049	−1.994022	5.830353
H	−1.282627	−3.223348	4.754648
H	−1.835353	−2.866786	2.350660

H	−0.706140	−1.240380	−1.880681
H	0.901344	−1.998477	−2.107940
H	0.953355	0.296781	−2.976144
H	0.730618	2.252975	−1.358720
H	0.106022	1.142636	−0.144064
H	−1.170169	1.745241	−2.890685
H	−1.826070	0.574673	−1.722861
H	−2.080664	2.374322	−0.022687
H	−1.446893	3.567832	−1.169424
H	−3.381783	3.085708	−2.736505
H	−4.013886	1.892342	−1.573199
H	−3.968676	3.621081	−1.141568
H	2.850306	1.214671	−1.722125
H	2.441341	0.249871	−0.292036
H	4.385632	−0.702895	−1.664179
H	3.054365	−1.858494	−1.472711
H	3.260214	−0.981460	−3.015310

Table S5. Optimized Cartesian coordinates in Å and ground-state RI-MP2 energies, $E_{\text{RI-MP2}}$, in atomic units (a.u.) for the $S_0 \leftarrow S_1$ MECP of $[\text{HS-H}]^-$ at the [RI-MP2/ADC(2)]/*aug*-cc-pVDZ level.

$E_{\text{RI-MP2}} = -845.6116187640$			
C	−0.247887	−0.329960	−0.522108
C	−1.098084	−1.336540	0.127760
C	−0.429837	−2.539406	0.616181
C	0.901318	−2.800290	0.345955
C	1.674069	−1.850590	−0.400559
C	1.114852	−0.653836	−0.823596
C	−0.733272	1.020127	−0.755219
O	−0.320778	1.518535	0.497218
O	−0.192683	1.620909	−1.960207
C	−0.239078	3.053554	−1.928577
C	−1.499893	3.576705	−1.224636
C	−1.566756	5.115903	−1.143286
C	−0.240530	5.668924	−0.585451
C	0.997219	5.169308	−1.340515
C	1.025513	3.635969	−1.283674
O	−2.360401	−1.228464	0.230180
C	−1.867618	5.725662	−2.523850
C	−2.695585	5.497644	−0.174612
C	2.275211	5.792037	−0.766494
H	−1.052372	−3.257199	1.160802
H	1.368759	−3.728718	0.694980
H	2.729990	−2.060428	−0.606296
H	1.700591	0.109487	−1.343396

H	−0.254691	3.355580	−2.995916
H	−1.489216	3.148470	−0.204092
H	−2.399501	3.195877	−1.741404
H	−1.124755	5.430731	−3.281009
H	−2.857901	5.386551	−2.873329
H	−1.882733	6.829669	−2.473369
H	−2.768784	6.594670	−0.061580
H	−3.667940	5.117478	−0.533496
H	−2.503036	5.055718	0.816746
H	−0.144623	5.351831	0.470842
H	−0.274820	6.777232	−0.591359
H	0.907999	5.485040	−2.398602
H	2.422623	5.461428	0.275747
H	3.165272	5.495245	−1.347764
H	2.216262	6.894930	−0.773717
H	1.059533	3.277057	−0.238524
H	1.912693	3.243156	−1.815259

Table S6. Optimized Cartesian coordinates in Å and ground-state RI-MP2 energies, $E_{\text{RI-MP2}}$, in atomic units (a.u.) for the $S_0 \leftarrow S_1$ MECP of $[\text{OS-H}]^-$ at the [RI-MP2/ADC(2)]/*aug*-cc-pVDZ level.

$E_{\text{RI-MP2}} = -807.5868736650$			
C	−0.440180	−1.036837	1.916487
C	−0.995299	0.064761	2.717543
C	−0.686192	0.078235	4.144023
C	−0.030426	−0.972417	4.758997
C	0.381110	−2.095888	3.972885
C	0.182750	−2.130854	2.598461
C	−0.409328	−0.955779	0.460228
O	0.766320	−0.204706	0.389170
O	−0.415963	−2.290784	−0.127205
C	−0.062078	−2.289145	−1.510803
C	1.466469	−2.325328	−1.804031
C	2.026625	−0.948572	−2.195571
C	1.613171	−0.513381	−3.604500
C	2.185167	0.853162	−3.995149
C	1.854454	1.228356	−5.445176
C	2.240669	−2.920820	−0.618142
C	1.827959	−4.362479	−0.296187
O	−1.765023	0.956542	2.239895
H	−1.042531	0.944447	4.711655
H	0.169624	−0.957383	5.836019
H	0.885894	−2.933716	4.467385
H	0.524904	−2.975763	1.995811
H	−0.527709	−1.413841	−2.004280

H	−0.535719	−3.199209	−1.924348
H	1.604456	−3.003502	−2.676192
H	3.133847	−0.984649	−2.155218
H	1.684741	−0.223767	−1.431912
H	1.951340	−1.270561	−4.341145
H	0.511366	−0.473203	−3.683635
H	1.789990	1.615366	−3.301720
H	3.281347	0.837505	−3.853992
H	2.262367	0.474085	−6.140608
H	0.761914	1.265145	−5.593606
H	2.272045	2.210003	−5.726185
H	3.320914	−2.890209	−0.859505
H	2.071566	−2.255290	0.244397
H	2.454542	−4.794953	0.504004
H	0.776629	−4.397353	0.031471
H	1.936082	−5.006962	−1.188033

S3. Schematic Structure of the $[HS\text{-}H]^-$ and $[OS\text{-}H]^-$ Chromophore

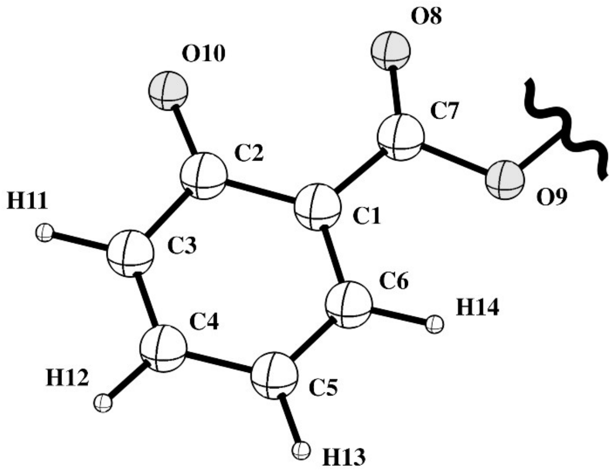

Scheme S1. Structure of the $[HS\text{-}H]^-$ and $[OS\text{-}H]^-$ chromophore. All atoms are labelled.
